# Variance, norms and cooperative behavior in public goods games

**DOI:** 10.3389/fpsyg.2024.1277707

**Published:** 2024-03-06

**Authors:** Guangrong Wang, Jianbiao Li, Wenhua Wang, Yue Wang, Jiafeng Wang

**Affiliations:** ^1^Neural Decision Science Laboratory, School of Economics and Management, Weifang University, Weifang, China; ^2^School of Economics, Institute for Study of Brain-like Economics, Shandong University, Jinan, China; ^3^Reinhard Selten Laboratory, China Academy of Corporate Governance, Nankai University, Tianjin, China; ^4^Deparment of Information Technology, Weifang Machinery Industry School, Weifang, China

**Keywords:** variance, reference point, empirical expectation, normative expectation, cooperative behavior

## Abstract

This study examines the relationship between the variance of others’ contributions, social norms (empirical and normative expectations), and cooperative behavior using a classic linear public goods game. The following results are observed. First, the variance of a participant’s group members’ contributions had a negative impact on their contributions, empirical expectations, and normative expectations. Second, deviations from the mean, whether negative or positive, were deemed less socially appropriate. Third, while there was a strong relationship between variance, social norms, and cooperative behavior, the mediating effect of social norms was found to be insignificant. Finally, there were some notable findings regarding behavior type. Although free riders and cooperators exhibited distinct behavioral patterns, their normative expectations were similar. Free riders expected others to cooperate, but their empirical expectations were significantly lower than cooperators’ expectations, which were aligned with their actual contributions. These findings contribute to research on the relationship between distribution heterogeneity, social norms and cooperative behavior. Furthermore, these findings provide valuable insights into management practices.

## 1 Introduction

Maintaining high levels of cooperation in social dilemmas has become a crucial concern since social dilemmas can be found in many domains, ranging from teamwork in organizations and charitable giving to the maintenance of public goods and environmental protection (Isaac and Walker, 1988; Sally, 1995; Chaudhuri, 2011; Gächter et al., 2017).

Public goods games are frequently employed to study cooperation in social dilemmas. Canonical findings from public goods game experiments show that while most people behave cooperatively, their contributions vary widely, ranging from zero to the entire endowment. Based on their cooperative behavior, people can be divided into different types, among which conditional cooperators are the most important type, that is, they contribute more when others contribute more (Fehr and Schmidt, 1999; Fischbacher et al., 2001; Fehr and Gächter, 2002; Fehr and Fischbacher, 2003, 2004; Ones and Putterman, 2007; Norenzayan and Shariff, 2008; Fischbacher and Gächter, 2010; Rustagi et al., 2010; Bigoni and Suetens, 2012; Camerer, 2013; Cheung, 2014; Hartig et al., 2015; Kuwabara and Yu, 2017; Fehr and Schurtenberger, 2018).

Conditional cooperators make cooperative decisions with reference to the contributions of their group members, as people generally evaluate things in relation to expectations or standards (Kempf and Ruenzi, 2006; Kőszegi and Rabin, 2006; Baillon et al., 2020; Cataldo and Cohen, 2020; Hashidate, 2021). Considering the heterogeneity of the contributions, a participant can utilize two significant references from other group members: the mean and the variance (heterogeneity). Because the average contribution can determine one’s monetary payoff and is easy to model, almost all theoretical models use it as a reference point, and previous empirical studies have also focused on reactions to average behavior. However, the literature on how variance affects cooperative behavior is surprisingly sparse.

In order to enhance and sustain cooperation, scholars seek to explore explanatory mechanisms for cooperative behavior. Social norms have been recognized as an important causal mechanism for cooperative behavior in social dilemmas (Fehr and Fischbacher, 2004; Reuben and Riedl, 2013; Fehr and Schurtenberger, 2018; Cabo et al., 2020; Quan et al., 2020; Kölle and Quercia, 2021). Based on the previous literature, by directly assuming the existence of a cooperation norm that a significant proportion of individuals have an intrinsic desire to follow, most of the regularities that violate the rationality assumption can be explained by social norms (Lindbeck et al., 1999; Ostrom, 2000; Krupka and Weber, 2013; Kimbrough and Vostroknutov, 2016; Fehr and Schurtenberger, 2018).

Some scholars explain social norms in terms of moral preferences, according to which people have preferences for following their norms. Many scholars have explored the mathematical foundations of moral preferences (see the review by Capraro and Perc, 2021). Xia et al. (2023) reviewed theoretical mechanisms of reciprocal cooperation from the perspective of reputation. Ugazio et al. (2022) explored the neuro-computational foundations of moral preferences, suggesting that human lives and money are valued in distinct neural currencies, supporting the theoretical proposal that human moral behavior is guided by processes that are distinct from those underlying behavior driven by personal material utility.

As the formation and maintenance of social norms are closely related to situational and personal factors, information about others’ contributions may impact cooperative norms (Titlestad et al., 2019). However, there is surprisingly little conclusive evidence on how such information affects social norms. To our knowledge, Kölle and Quercia (2021) investigated the impact of the mean on social norms, but no studies have examined how variance influences social norms. If variance has an impact on cooperative behavior, does it affect cooperative behavior directly or does it influence social norms which, in turn, affect cooperative behavior? To address this question, the study utilizes a classic public goods game to examine the relationship between variance, social norms and cooperative behavior.

While social norms are commonly defined as standards of behavior that indicate how individuals ought to behave in a given situation, the definitions based on social expectations are widely accepted (Elster, 1989; Fehr and Fischbacher, 2004; Bicchieri, 2006, 2016). Most prominently, as defined by Bicchieri (2006, 2016), social norms are behavioral rules that individuals prefer to conform to on condition that they believe that (i) most people in their reference network conform to it (empirical expectation), and (ii) most people in their reference network believe that they ought to conform to it (normative expectation). Empirical expectations are beliefs about what other people will do in certain situations, while normative expectations are beliefs about what other people believe ought to be done. In some literature, empirical expectations are called descriptive social norms, while normative expectations are called injunctive social norms (Rivis and Sheeran, 2003; Schultz et al., 2007; Bicchieri, 2016). Following these studies, we seek to investigate the relationship between variance, empirical and normative expectations, and cooperative behavior^1^.

This study designed three experimental treatments to measure the cooperative behavior, empirical expectations, and normative expectations: a choice treatment, an empirical expectations treatment, and a normative expectations treatment. We used a classic linear public goods game with four members for each group, and the conditional contribution version of this game was applied. This paper wants to examine the impact of variance of the other group members’ contributions on social norms and cooperative behavior. To facilitate comparison, three distributions with the same mean but different variances were exogenously established. Without loss of generality, three distributions with a mean of 10 MU were selected. The corresponding standard deviations were zero (labeled Z-variance), 6 (labeled M-variance), and 9.5 (labeled L-variance), respectively. In the empirical and normative treatments, participants reported their empirical and normative expectations for each decision situation as spectators. To induce empirical expectations, we elicited incentivized beliefs about participants’ actual behavior in the choice experiment. To induce normative expectations, all spectators were asked to evaluate the social appropriateness of all given actions.

## 2 Materials and methods

### 2.1 Participants

A total of 120 participants (mean age: 21.33 years; 58 males, 62 females) who had never participated in a public goods experiment were recruited to take part in one session each. Each session lasted around 50 min. All experiments were conducted at the Institute for Study of Brain-like Economics, Shandong University, China. All participants signed informed consent prior to the experiment, which was performed in accordance with the Declaration of Helsinki and was approved by the Ethics Committee of College of Economics, Shandong University. The study is not pre-registered. The experiment was programmed and conducted in z-Tree (Fischbacher, 2007). The mean monetary reward was 46 Chinese Yuan ($ 6.76).

### 2.2 Experiment design

The experimental setting was a standard linear public goods game with four members for each group. Each group member was endowed with 20 MU (Monetary Units) and had to decide either to keep 20 MU or to contribute to a group project with a fraction of their endowment in the range of 0–20 MU. The payoff function is given as Equation (1)


(1) $$\pi_{i}=20-g_{i}+0.5\sum_{j=1}^{4}g_{j},$$

where $g_{i}$ is the contribution of participant *i* and $g_{j}$ is the contribution of each group member. The amount contributed to the project was doubled and was shared equally among the four group members. While the contribution of each MU was worth 0.5 MU to each group member, i.e., 2 MU to the group, the amount of each MU kept for oneself was worth 1 MU to the participant. Therefore, according to the standard assumption, all participants would contribute zero, i.e., $g_{j}=0$ for all *j*. This created the classic free rider problem and resulted in socially inefficient outcomes.

**Figure 1:**
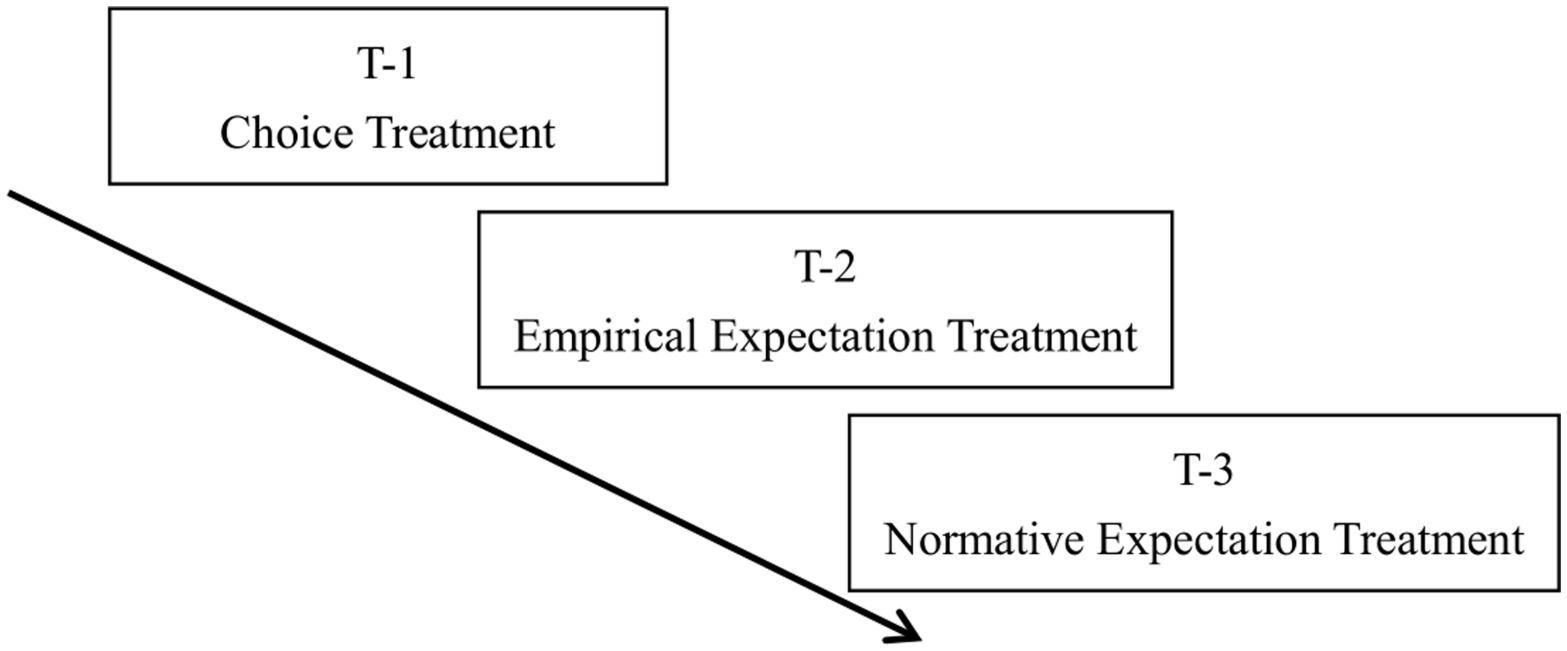
Experimental flow-chart. In T-1, we measured cooperative behavior for three conditions (zero, medium, and large variances). In T-2, we elicited empirical expectations on each of the three conditions. In T-3, we elicited normative expectations on each possible contribution of the three conditions. We applied a within-subjects design where the participants completed each condition of all treatments.

We designed three experimental treatments: a choice treatment, an empirical expectation treatment and a normative expectation treatment. In each treatment, there were three levels of variance (zero, medium, and large variance), constituting three conditions. We implemented a within-subjects design^2^, whereby each participant made one decision per condition in all treatments (Figure 1). The treatments were not counterbalanced because our objective was to first classify the participants based on their behavior in the conditional decisions, before examining the connections between variance, norms and behavior.

### 2.3 Choice treatment

A variant of the so-called “strategy method” was applied to elicit participants’ cooperative preferences (Fischbacher et al., 2001; Fischbacher and Gächter, 2010; Burton-Chellew et al., 2016). This method is capable to observe contributions as a function of other group members’ contributions without using deception (Fischbacher et al., 2001). For conditional contributions, participants had to decide how much they would contribute to the group project, given possible distributions of the other three group members. Specifically, a “contribution table” of the three possible distributions of the other three group members (10, 10, 10; 5, 8, 17; 0, 11, 19) was shown, and participants had to make their corresponding contributions for each of the three distributions. The experiment was conducted only once, and the participants were aware of this. Thus, participants’ preferences were elicited without mixing preferences with strategic considerations.

### 2.4 Empirical expectation treatment

To elicit empirical expectations, a participant was asked to report his/her belief on the actual behavior of participants in the choice treatment by guessing their average contributions conditional on each of the three possible distributions. To incentivize participants to express their true beliefs, a binarized scoring rule was used (Hossain and Okui, 2013). That is, the closer their guess was to the average contribution of all participants, the higher the probability of winning the 20 MU prize. At the end of the experiment, a situation was randomly selected, and each participant’s guess in that scenario would determine their prize.

### 2.5 Normative expectation treatment

The well-established paradigm of Krupka and Weber (2013) was used to elicit normative expectations. In our experimental task, participants had to evaluate the social appropriateness of others’ actions on a six-point scale ranging from 1: “very socially inappropriate” to 6: “very socially appropriate.” For each of the three possible distributions of other group members, participants were asked to evaluate how socially appropriate they thought it was to contribute c∈[0, 5, 10, 15, 20] MU. We limited the evaluation of actions to these five cases to reduce the workload on the participants and to avoid random behavior due to boredom.

The incentive mechanism for eliciting normative expectations was as follows. At the end of the experiment, a situation was randomly selected, and the participant’s response in that scenario was compared with that of all other participants. If a participant’s appropriateness rating matched the modal response, they would earn 20 MU; otherwise, they would earn nothing. This incentive mechanism encourages participants to reveal their true perceptions of what is commonly regarded as appropriate or inappropriate behavior. This is necessary because social norms are collectively recognized rules of behavior, rather than personal opinions about behavior (Elster, 1989; Ostrom, 2000; Krupka and Weber, 2013; Kölle and Quercia, 2021).

## 3 Behavioral predictions

Based on the direct social norms approach, a norm $c^{*}$, to which people have an inherent desire to conform, is defined in terms of a particular behavior. In our context of cooperation, $c^{*}$ describes a level of cooperation that is consistent with the normative expectation. Formally, following Fehr and Schurtenberger (2018), a participant’s utility function $u_{i}$ is given by Equation (2)


(2) $$\;u_{i}=\left\{\begin{array}{c} \pi_{i}\left(x_{i},x_{-i}\right)-\gamma_{i}{\left(x_{i}-c^{*}\right)}^{2}\;\text{if}\;x_{i}<c^{*}\\ \pi_{i}\left(x_{i},x_{-i}\right)\;\text{if}\;x_{i}\geq c^{*} \end{array}\right.$$

The $\text{term}\;\pi_{i}\left(x_{i},x_{-i}\right)$ denotes individual *i’s* material payoff, $x_{i}$ is his/her contribution, and $\gamma_{i}{\left(x_{i}-c^{*}\right)}^{2}$ denotes the psychological cost of deviating from the social norm $c^{*}$ for the cooperators. The term $\gamma_{i}\geq 0$ captures an individual’s strength of the desire to comply with the norm.

In our setting, the average contribution of the other three members in a group is 10 MU, except for different variance. Therefore, the utility function $u_{i}$ is described by Equation (3)


(3) $$\begin{array}{l} \;u_{i}=\frac{2\times \left(3\times 10+x_{i}\right)}{4}+\left(20-x_{i}\right)-\gamma_{i}{\left(x_{i}-c^{*}\right)}^{2}\\ \;=35-\frac{x_{i}}{2}-\gamma_{i}{\left(x_{i}-c^{*}\right)}^{2} \end{array}$$

$$\;u_{i}^{\prime }=-\frac{1}{2}-2\gamma_{i}\left(x_{i}-c^{*}\right)=0$$

(4) $$\;x_{i}=-\frac{1}{4\gamma_{i}}+c^{*}$$

Therefore, the utility maximization contribution of a conditional cooperator depends on the norm $c^{*}$ and their desired degree $\gamma_{i}$ to comply with the norm. Both $c^{*}$ and $\gamma_{i}$ positively affect their contributions. Therefore, Hypothesis 1 is proposed.

*Hypothesis 1*: Social norms have a positive effect on cooperation.

In our setting, the three distributions have the same mean but different variances. For each distribution, the norm $c^{*}$ should be different. The mean must be an important factor affecting the norms, since it can determine the monetary payoff (Fehr and Schurtenberger, 2018; Kölle and Quercia, 2021). Because previous research has found that people are more likely to follow the bad example than the good example (De Oliveira et al., 2015; Hartig et al., 2015; Irlenbusch et al., 2019), minimal contributions from other group members were more likely to influence the participants’ contributions. Moreover, the F-S model suggests that in public goods games, a player never contributes more than the minimum of the other group members’ contributions (Fehr and Schmidt, 1999). Therefore, we establish a norm $c^{*}$ for the three distributions.


(5) $$\begin{array}{l} \;c_{j}^{*}=\alpha \times {\text{mean}}_{j}+\beta \times {\text{minimum}}_{j}\;\\ j=\text{zero},\text{medium},\text{large variance} \end{array}$$

The term ${\text{mean}}_{j}$ refers to the mean, and ${\text{minimum}}_{j}$ refers to the minimum contribution of each distribution. For the distributions with zero, medium, and large variances, the social norms ($c_{j}^{*}$) should be 10 (α+β),10α+5β and 10 α, respectively. Obviously, $c_{\text{zero}}^{*}>c_{\text{medium}}^{*}>c_{\text{large}}^{*}$. Thus, Hypothesis 2 is proposed.

*Hypothesis 2*: Given the same mean, the variance of others’ contributions has a negative effect on the social norms.

In classic public goods games, the contributions of a participant’s group members can provide two important reference points: the mean and the variance of other members’ contributions. This study examines the impact of variance on cooperative behavior by examining three distributions with the same mean but varying variances. Based on Equation (5), the variance of others’ contributions can influence participants’ social norms, and based on Equation (4), social norms can affect their cooperative behavior. Therefore, Hypothesis 3 is proposed.

*Hypothesis 3*: Social norms play a mediating role in the relationship between variance and cooperative behavior.

## 4 Results

### 4.1 Effect of variance on cooperative behavior

The results of the repeated measures ANOVA showed a significant main effect of variance on the conditional contributions [*F*(2, 238) = 16.461, *p* < 0.001, *η_p_^2^* = 0.122]^3^. The mean contributions in the zero, medium and large variance conditions are shown in Table 1. The mean contributions in the zero variance condition were larger than those in the medium and large variance conditions [Paired-*t* test: *T*^4^ = 3.238, *p* = 0.002, Cohen’s *d* = 0.30, 95% CI: 0.112, 0.478; *T* = 4.617, *p* < 0.001, Cohen’s d = 0.42, 95% CI: 0.234, 0.607], and the mean contributions in the medium variance condition were larger than those in the large variance condition [*T* = 3.756, *p* < 0.001, Cohen’s *d* = 0.33, 95% CI: 0.142, 0.509].

**Table 1:** Mean contributions.

Behavior type	Variance	Mean (MU)	Standard error
All	Zero	5.23	0.499
	Medium	4.21	0.466
	Large	3.35	0.476
Cooperators	Zero	9.09	0.491
	Medium	7.32	0.571
	Large	5.83	0.692
Free rides	Zero	0.00	0.000
	Medium	0.00	0.000
	Large	0.00	0.000

Consistent with previous studies, our participants can also be divided into free-riders, who always contribute zero, and cooperators, who consider the interests of others. 42.5% of the 120 participants were free-riders, and 57.5% were cooperators who contributed some money in any or all conditions.

For the cooperators, the results of the repeated measures ANOVA showed a significant main effect of variance on conditional contributions [*F*(2, 238) = 18.221, *p* < 0.001, *η_p_^2^* = 0.211]. The average contributions in the zero variance condition were greater than those in the medium and large variance conditions [*T* = 3.338, *p* = 0.001, Cohen’s *d* = 0.40, 95% CI: 0.155, 0.646; *T* = 4.941, *p* < 0.001, Cohen’s *d* = 0.60, 95% CI: 0.337, 0.849], and the average contributions in the medium variance condition were greater than those in the large variance condition [*T* = 3.716, *p* < 0.001, Cohen’s *d* = 0.45, 95% CI: 0.198, 0.693].

### 4.2 Effect of variance on empirical expectations

The results of the repeated measures ANOVA showed a significant main effect of the variance of other group members’ contributions on their empirical expectations [*F*(2, 238) = 35.675, *p* < 0.001, *η_p_^2^* = 0.231] for all participants. Low heterogeneity of other members’ contributions led to higher empirical expectations compared to medium and high heterogeneity (Table 2). The empirical expectations in the zero variance condition were greater than those in the medium and large variance conditions [*T* = 4.621, *p* < 0.001, Cohen’s *d* = 0.42, 95% CI: 0.234, 0.608; *T* = 6.892, *p* < 0.001, Cohen’s *d* = 0.63, 95% CI: 0.432, 0.824], and the empirical expectations in the medium variance condition were greater than those in the large variance condition [*T* = 5.093, *p* < 0.001, Cohen’s *d* = 0.46, 95% CI: 0.276, 0.652].

**Table 2:** Mean empirical expectations.

Behavior type	Variance	Mean (MU)	Standard error
All	Zero	7.22	0.369
	Medium	5.88	0.368
	Large	4.24	0.437
Free riders	Zero	5.67	0.617
	Medium	4.35	0.556
	Large	1.94	0.520
Cooperators	Zero	8.36	0.404
	Medium	7.01	0.447
	Large	5.94	0.578

**Figure 2:**
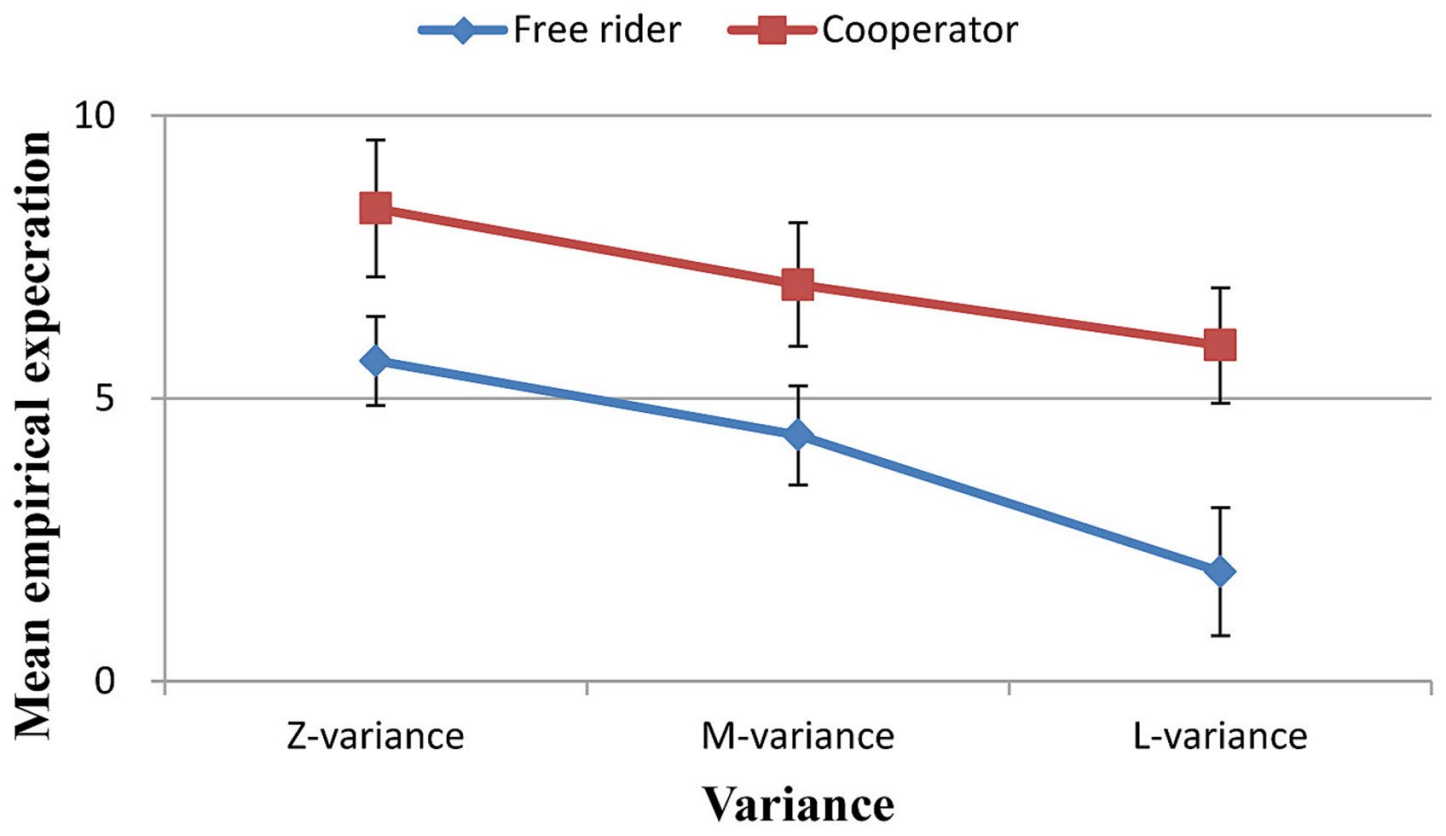
Empirical expectations for different variances.

For the free riders, the results of the repeated measures ANOVA showed a significant main effect of the variance of other group members’ contributions on their empirical expectations [*F*(2, 238) = 24.768, *p* < 0.001, *η_p_^2^* = 0.331]. The empirical expectations in the zero variance condition were greater than those in medium and large variance conditions [*T* = 3.93, *p* < 0.001, Cohen’s *d* = 0.55, 95% CI: 0.253, 0.843; *T* = 5.80, *p* < 0.001, Cohen’s *d* = 0.81, 95% CI: 0.492, 1.126], and the empirical expectations in the medium variance condition were greater than those in the large variance condition [*T* = 4.133, *p* < 0.001, Cohen’s *d* = 0.58, 95% CI: 0.279, 0.873] (Figure 2).

For the cooperators, the results of the repeated measures ANOVA showed a significant main effect of the variance of other group members’ contributions on their empirical expectations [*F*(2, 238) = 13.687, *p* < 0.001, *η_p_^2^* = 0.168]. The empirical expectations in the zero variance condition were greater than those in the medium and large variance conditions [*T* = 3.071, *p* = 0.003, Cohen’s *d* = 0.37, 95% CI: 0.124, 0.612; *T* = 4.199, *p* < 0.001, Cohen’s *d* = 0.51, 95% CI: 0.253, 0.755], and the empirical expectations in the medium variance condition were greater than those in the large variance condition [*T* = 3.097, *p* = 0.003, Cohen’s *d* = 0.37, 95% CI: 0.127, 0.616] (Figure 2).

Furthermore, the empirical expectations of the free riders were lower than those of the cooperators in any variance level (Figure 2; Table 3).

**Table 3:** Comparison of empirical expectations for different behavior type.

Variance	*T*	*p*	Cohen’s *d*	95% Confidence Interval	
				Lower	Upper
Zero	3.81	<0.001	0.70	0.313	1.09
Medium	3.77	<0.001	0.70	0.306	1.08
Large	4.95	<0.001	0.92	0.507	1.31

### 4.3 Effect of variance on normative expectations

Following the approach of Krupka and Weber (2013), mean appropriateness ratings were calculated by transforming participants’ responses into evenly spaced numerical scores using the following scale: very socially inappropriate = −1; inappropriate = −0.6; somewhat socially inappropriate = −0.2; somewhat socially appropriate = 0.2; socially appropriate = 0.6; very socially appropriate = 1.

A 5 (contribution: 0, 5, 10, 15, 20) × 3 (variance: zero, medium, large) ANOVA showed significant main effects of variance [*F*(2, 238) = 24.933, *p* < 0.001, *η_p_^2^* = 0.173], contribution [*F*(4, 476) = 51.754, *p* < 0.001, *η_p_^2^* = 0.303] on normative expectations in the certainty decision. A significant contribution × variance interaction effect on normative expectations was observed [*F*(4, 476) = 42.235, *p* < 0.001, *η_p_^2^* = 0.262].

**Figure 3:**
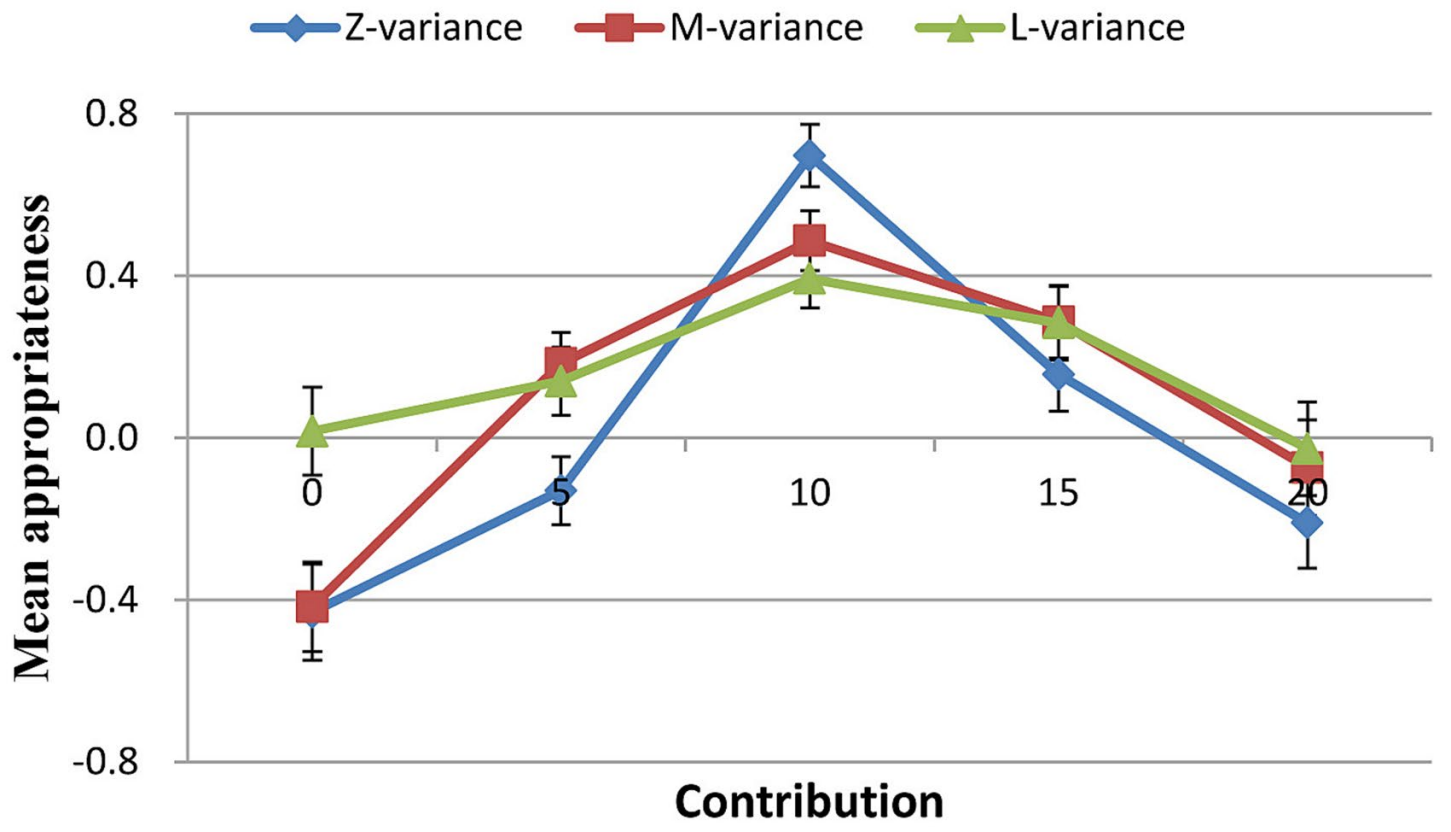
Social appropriateness of conditional contributions.

For all three distributions, contributing 10 MU (i.e., the mean contribution) was considered the most socially appropriate behavior. Mean social appropriateness ratings in the zero variance condition were higher than those in the medium variance condition (*T* = 6.039, *p* < 0.001, Cohen’s *d* = 0.55, 95% CI: 0.358, 0.742), which were higher than those in the large variance condition (*T* = 3.556, *p* = 0.001, Cohen’s *d* = 0.32, 95% CI: 0.140, 0.508) (Figure 3; Table 4).

**Table 4:** Normative expectations for free riders and cooperators.

Type	Variance	Free rider		Cooperator		*T*	*p*	Cohen’s *d*	95% CI	
		Mean	SE	Mean	SE				Lower	Upper
10 MU contribution	Zero	0.58	0.072	0.79	0.040	−2.696	0.008	−0.498	−0.870	−0.121
	Medium	0.40	0.067	0.55	0.042	−2.099	0.038	−0.388	−0.756	−0.016
	Large	0.29	0.063	0.47	0.043	−2.331	0.021	−0.430	−0.800	−0.057
20 MU contribution	Zero	−0.32	0.091	−0.13	0.072	−1.632	0.105	−0.301	−0.667	0.067
	Medium	−0.11	0.091	−0.04	0.081	−0.575	0.567	−0.106	−0.468	0.257
	Large	−0.11	0.090	0.03	0.078	−1.158	0.249	−0.214	−0.577	0.152
0 MU contribution	Zero	−0.19	0.097	−0.61	0.070	3.536	0.001	0.653	0.266	1.034
	Medium	−0.21	0.091	−0.57	0.066	3.328	0.001	0.614	0.230	0.993
	Large	0.15	0.086	−0.08	0.070	2.027	0.045	0.374	0.003	0.742

**Figure 4:**
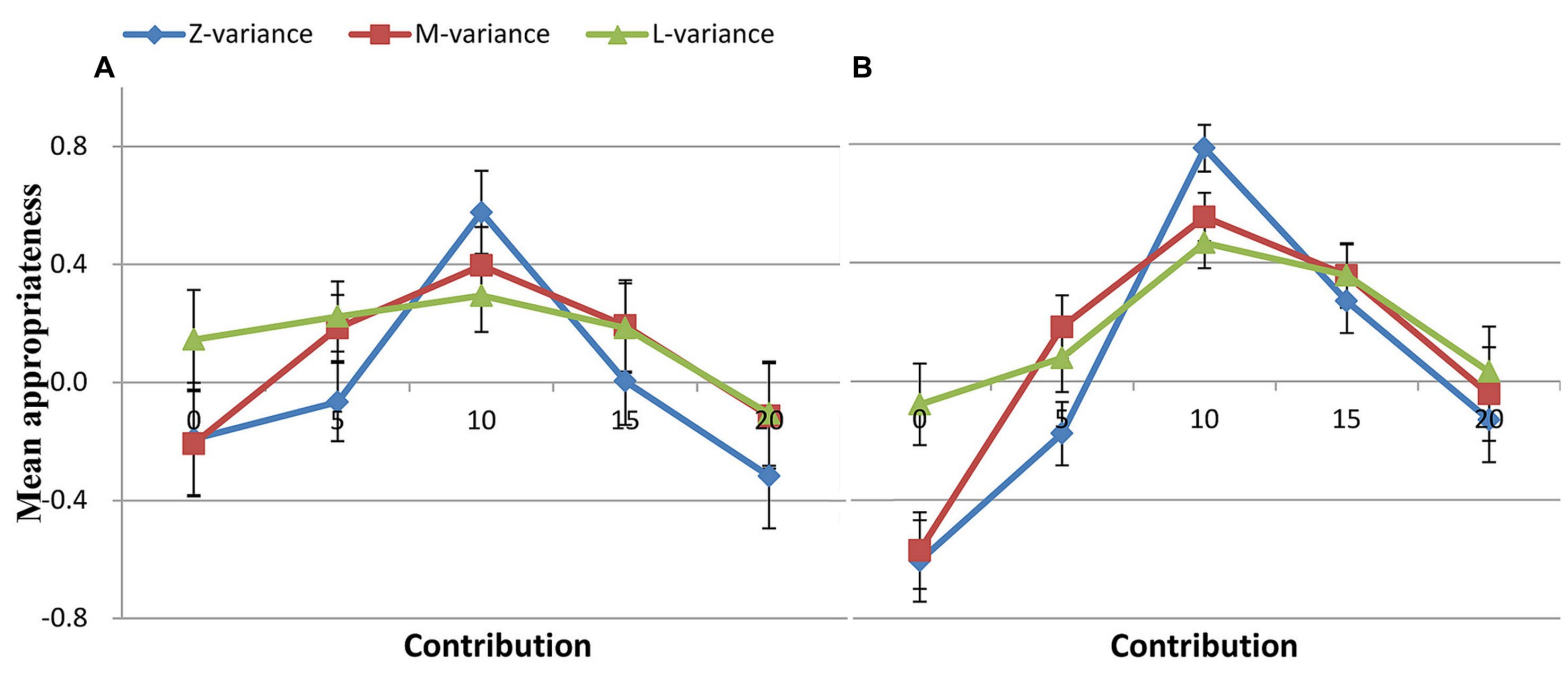
Social appropriateness of conditional contributions for different types. **(A)** Free rider, **(B)** Cooperator.

For the free riders, a 5 (contribution: 0, 5, 10, 15, 20) × 3 (variance: zero, medium, large) ANOVA showed significant main effects of variance [*F*(2, 238) = 12.980, *p* < 0.001, *η_p_^2^* = 0.206], contribution [*F*(4, 476) = 10.922, *p* < 0.001, *η_p_^2^* = 0.179] on normative expectations. A significant contribution × variance interaction effect on normative expectations was observed [*F*(4, 476) = 14.176, *p* < 0.001, *η_p_^2^* = 0.221] (Figure 4A). For the cooperators, a 5 (contribution: 0, 5, 10, 15, 20) × 3 (variance: zero, medium, large) ANOVA showed significant main effects of variance [*F*(2, 238) =12.488, *p* < 0.001, *η_p_^2^* = 0.155], contribution [*F*(4, 476) =56.102, *p* < 0.001, *η_p_^2^* = 0.452] on normative expectations. A significant contribution × variance interaction on normative expectations was observed [*F*(4, 476) =30.664, *p* < 0.001, *η_p_^2^* = 0.311] (Figure 4B).

Although both free riders and cooperators exhibited comparable patterns in all three conditions, cooperators’ ratings were higher than free riders’ for the contribution of 10 MU (Figure 4; Table 4). Interestingly, the rating pattern for positive and negative deviations showed an opposite trend for cooperators and free riders. For instance, although it was not statistically significant, for the contribution of 20 MU, cooperators’ ratings were higher than those of free riders. Conversely, for the contribution of 0 MU, cooperators’ ratings were significantly lower than those of free riders. But whether cooperators or free riders, what was considered most socially appropriate was to contribute the mean.

We conducted a sensitivity power analysis using G*power 3.1 (Faul et al., 2007). For the repeated measures ANOVAs, we set the parameters as follows: α error probability = 0.05; power = 0.8, total sample size = 120; number of groups = 1; number of measurements = 3; and non-sphericity correction, ϵ = 1. The results indicate that the effect size is 0.116. Regarding our ANOVAs on empirical expectations, the smallest *η_p_^2^* is 0.168, i.e., the effect size is *f* = 0.45, which is larger than the effect size value derived through with sensitivity analysis (0.116). Based on our ANOVAs on normative expectations, the smallest *η_p_^2^* is 0.155, i.e., the effect size is *f* = 0.43, which is larger than the effect size value obtained through sensitivity analysis (0.116). For the t-tests, we set the parameters as follows: α error probability = 0.05; power = 0.8, total sample size = 120. The results of the sensitivity analysis indicate that Cohen’s *d* is 0.25. For the results of *t*-tests on empirical and normative expectations, Cohen’s *d*s of all T-statistics are greater than 0.25. These findings demonstrate the impact of variance on empirical and normative expectations, so our results support Hypothesis 2 (i.e., given the same mean, the variance of others’ contributions has a negative effect on the social norms).

### 4.4 Relationship between social norms and cooperative behavior

**Figure 5:**
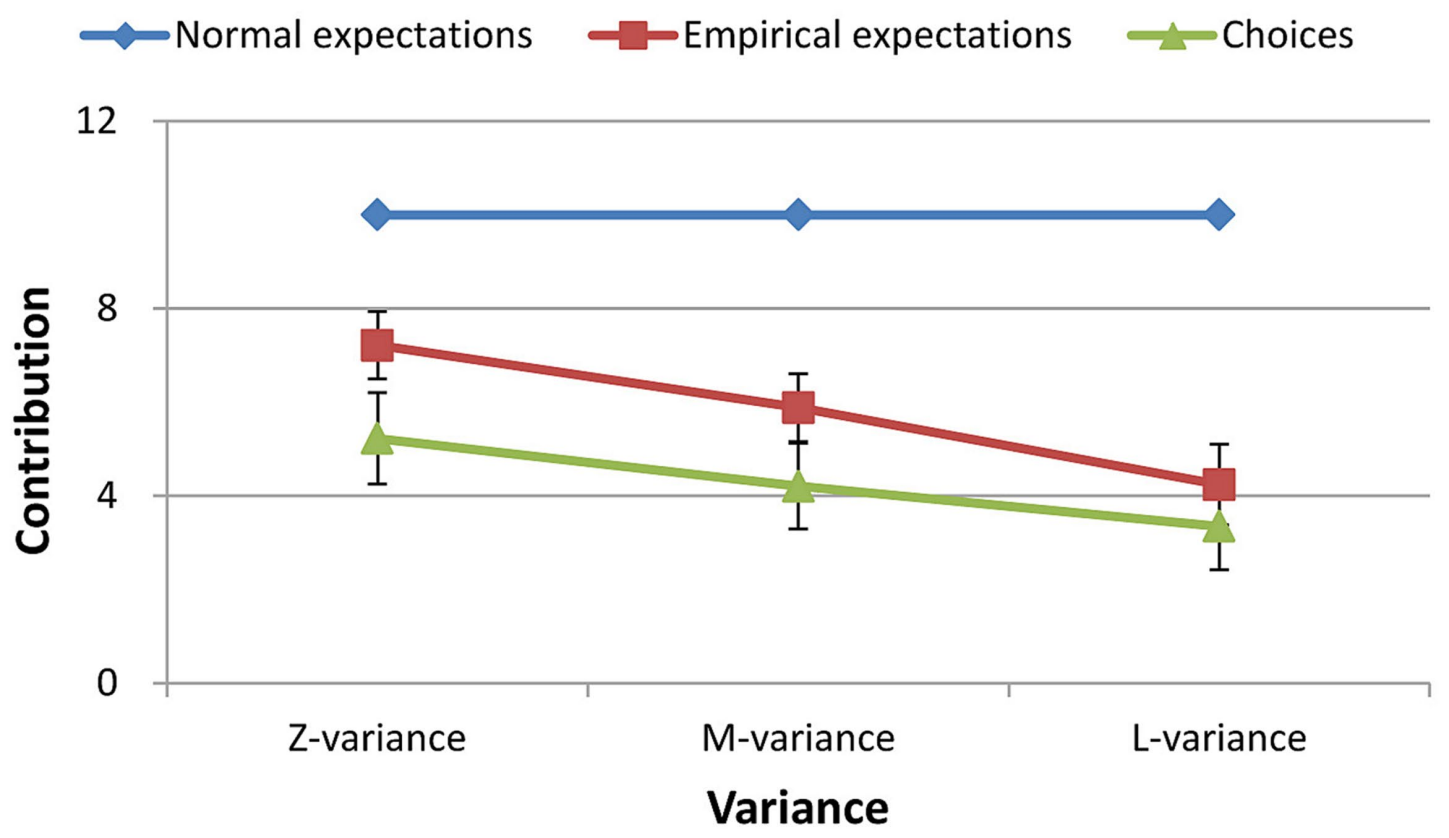
Empirical expectations, normative expectations, and cooperation behavior.

We combine the results from conditional contributions, empirical expectations, and normative expectations into a single graph to compare the overall relationship between contributions and social norms (Figure 5). The blue diamonds depict the contributions considered most socially appropriate, the red squares display mean empirical expectations, and the green triangles represent average contributions.

In all three conditions, the mean contributions were lower than the mean empirical expectations (Tables 1, 2; Figure 5), which were lower than the normative expectations (paired *t*-test: all *p* < 0.01 except for contributions and empirical expectations in the large variance condition, Table 5) for all participants. These results indicate a tendency for individuals to overvalue the contributions of others or to contribute less than others in order to gain more personal benefits. However, they thought that an individual ought to contribute equally. Although they expected that not everyone would adhere to their normative expectations, they underestimated the degree of noncompliance. That is, actual contributions were significantly less than empirical expectations in all cases.

**Table 5:** Comparisons between empirical expectations and contributions.

Type	Variance	*T*	*p*	Cohen’s *d*	95% Confidence interval	
					Lower	Upper
All	Zero	−4.07	<0.001	−0.372	−0.556	−0.186
	Medium	−3.85	<0.001	−0.351	−0.535	−0.166
	Large	−1.88	0.062	−0.172	−0.352	0.009
Free riders	Zero	−9.19	<0.001	−1.287	−1.656	−0.911
	Medium	−7.83	<0.001	−1.097	−1.442	−0.745
	Large	−3.74	<0.001	−0.523	−0.814	−0.228
Cooperators	Zero	1.40	0.165	0.169	−0.069	0.406
	Medium	0.58	0.563	0.070	−0.167	0.306
	Large	−0.16	0.872	−0.019	−0.255	0.217

**Figure 6:**
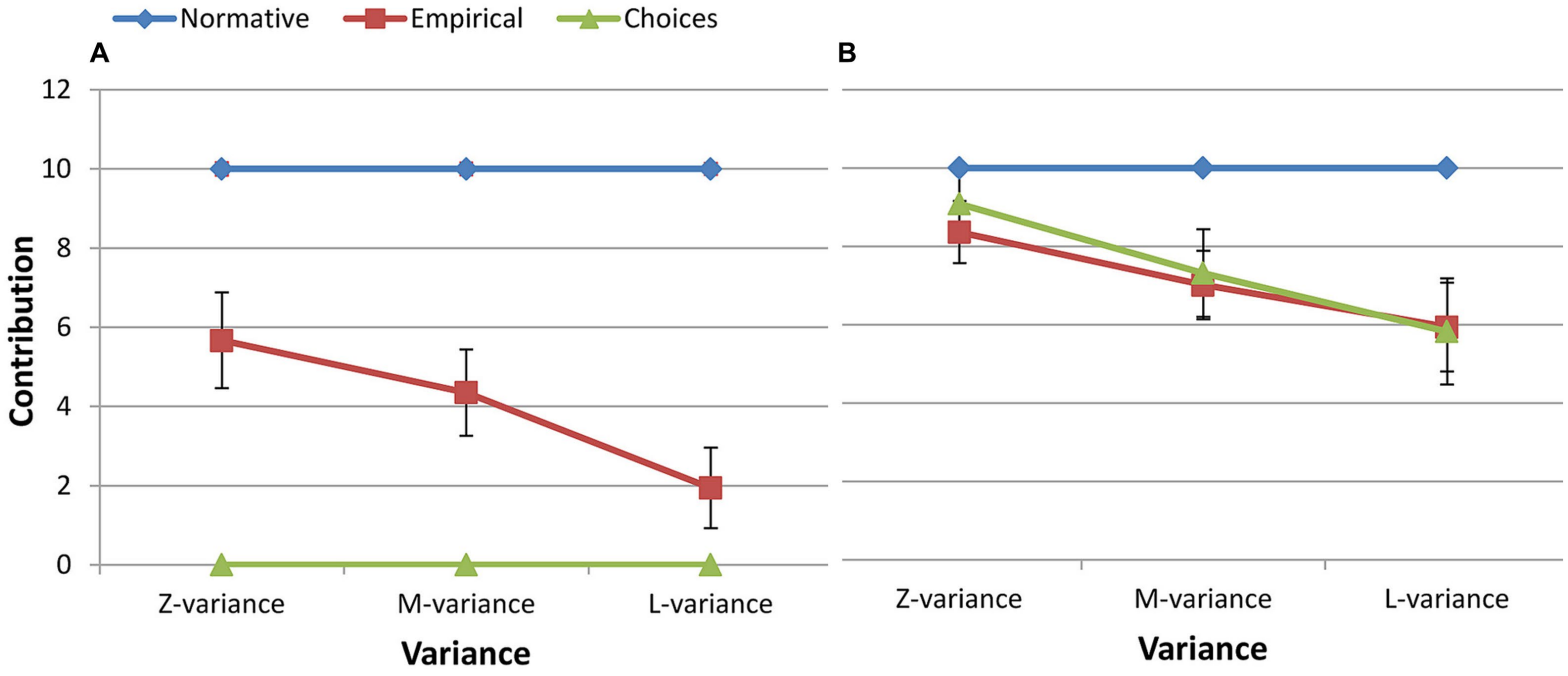
Empirical expectations, normative expectations, and cooperation behavior. **(A)** Free rider, **(B)** Cooperator.

The free riders consistently contributed 0 MU, but they held the belief that others would contribute more than they did. As a result, their empirical and normative expectations were significantly higher than 0, while their normative expectations exceeded their empirical expectations in all cases (Table 5; Figure 6). The cooperators, on the other hand, did not display a significant deviation between their actual contributions and their empirical expectations (Table 5; Figure 6). This indicates that not only did they predict that not everyone would conform to social norms, but they also correctly estimated the degree of compliance.

In general, both free riders and cooperators believed that it was the social norm to contribute the same amount as the average of others, but their empirical expectations differed. Free riders overestimated the degree of compliance of their peers and underestimated that of cooperators, whereas cooperators correctly estimated the degree of compliance of their peers but overestimated that of free riders.

We conducted a general linear model analysis to examine the relationship between social norms and cooperative behavior. The results showed that social norms can predict the level of contributions [empirical expectations: *F*(15, 319) *=* 12.49, *p <* 0.001, *η_p_^2^* = 0.763; normative expectations: *F*(5, 343) = 4.99, *p =* 0.009, *η_p_^2^* = 0.655]. Based on the sensitivity power analysis above, the effect sizes of empirical and normative expectations are larger than the effect size value obtained through the sensitivity analysis. Thus, our results support Hypothesis 1 (i.e., social norms have a positive effect on cooperation).

### 4.5 Mediation analysis

Based on our results, variance influences contributions, empirical and normative expectations, while empirical and normative expectations influence cooperation. Therefore, we aim to examine whether social norms act as mediators in the relationship between these three factors.

Because we conducted a within-subjects design, in which data were measured repeatedly within individuals in different conditions, the usual between-subjects mediations do not seem to fit our within-subjects data (Preacher and Hayes, 2008; Zhao et al., 2010; Hayes, 2013). Therefore, we conducted a Bayesian multilevel mediation analysis using the bmlm package in R to build and fit the mediation model (Vuorre and Bolger, 2018). This mediation model is appropriate for variables that are repeatedly measured within individuals. We used variance as the independent variable (IV), empirical expectation and normative expectation as mediators (M), and contribution as the outcome variable (DV) in the mediation. To ensure stable results, we increased the number of iterations from the default of 2000 to 10,000 for the MCMC sampler. We conducted two mediation analyses with empirical expectations and normative expectations as moderators, respectively.

**Figure 7:**
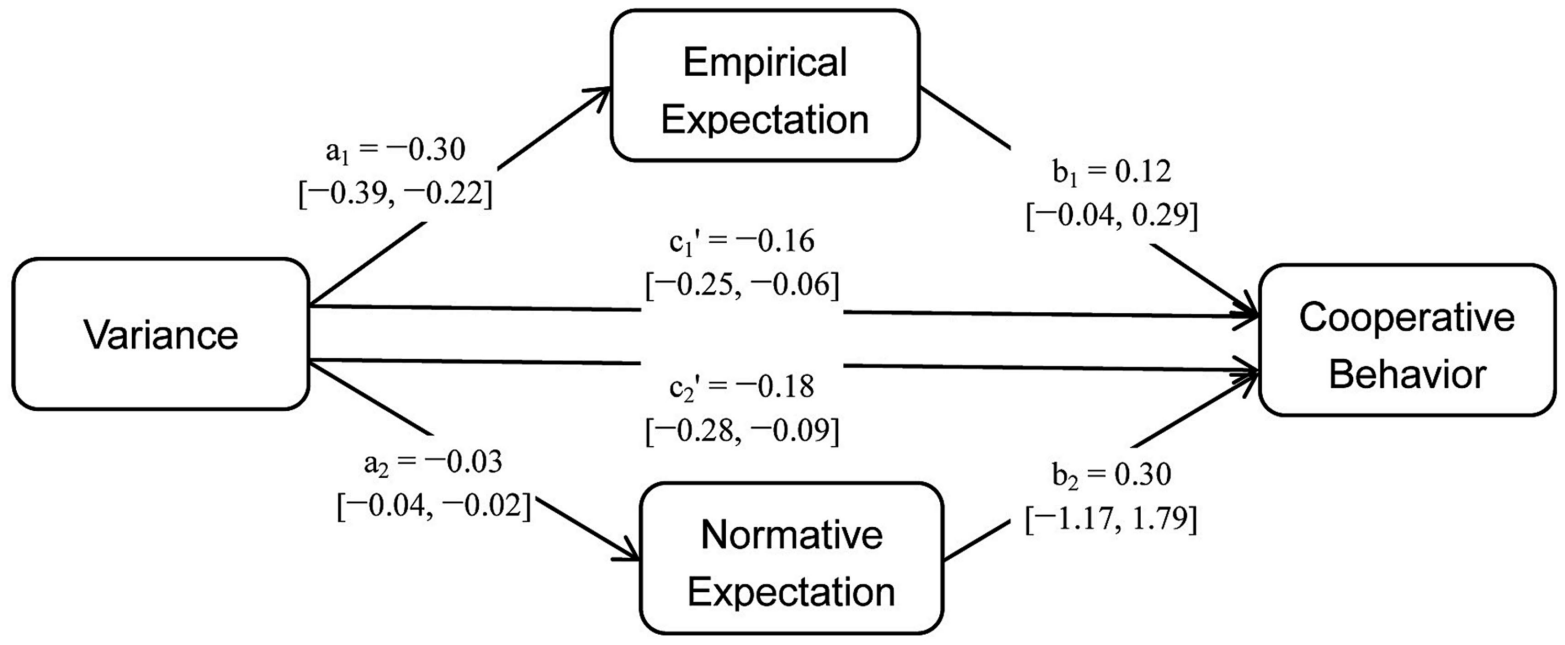
Conceptual models with coefficient estimation results.

Table 6 presents the results of multilevel mediation analysis for empirical expectations. Although both the total effect (c_1_ = −0.19, 95% CI: −0.28, −0.11) and direct effect (c_1_′ = −0.16, 95% CI: −0.25, −0.06) were significant, the mediation effect of empirical expectations (a_1_ × b_1_ = −0.04, 95% CI: −0.10, 0.02) was not and the proportion of the effect that was mediated was only 0.19 (95% CI: −0.10, 0.53). Table 6; Figure 7 show that variance had a stronger influence on contributions (path c_1_′) than empirical expectations (path b_1_). This means that participants used variance as a direct cue to make their contributions.

**Table 6:** Mediation effect of empirical expectations.

Parameter	Mean	SE	Median	2.5%	97.5%
Effect of IV on M (a_1_)	−0.30	0.04	−0.30	−0.39	−0.22
Effect of M on DV (b_1_)	0.12	0.09	0.12	−0.04	0.29
Direct Effect (c_1_′)	−0.16	0.05	−0.16	−0.25	−0.06
Indirect Effect (a_1_ × b_1_)	−0.04	0.03	−0.04	−0.10	0.02
Total Effect (c_1_)	−0.19	0.04	−0.19	−0.28	−0.11
pme	0.19	0.16	0.19	−0.10	0.53

IV, Independent Variable (Variance); M, Mediating Variable (Empirical Expectation); DV, Dependent Variable (Contribution). SE (for Standard Error) is the posterior standard deviation. pme is the proportion of effect that is mediated.

Similarly, Table 7 presents the results of multilevel mediation analysis for normative expectations. Both the total effect (c_2_ = −0.19, 95% CI: −0.28, −0.11) and direct effect (c_2_′ = −0.18, 95% CI: −0.28, −0.09) of variance on contributions were significant, while the mediation effect of normative expectations (a_2_ × b_2_ = −0.01, 95% CI: −0.06, 0.04) was not significant, and the proportion of the effect that was mediated was only 0.05 (95% CI: −0.24, 0.32). Table 7; Figure 7 show that variance had a stronger influence on contributions (path c_2_′) than normative expectations (path b_2_).

**Table 7:** Mediation effect of normative expectations.

Parameter	Mean	SE	Median	2.5%	97.5%
Effect of IV on M (a_2_)	−0.03	0.00	−0.03	−0.04	−0.02
Effect of M on DV (b_2_)	0.30	0.76	0.30	−1.17	1.79
Direct Effect (c_2_′)	−0.18	0.05	−0.18	−0.28	−0.09
Indirect Effect (a_2_ × b_2_)	−0.01	0.03	−0.01	−0.06	0.04
Total Effect (c_2_)	−0.19	0.04	−0.19	−0.28	−0.11
pme	0.05	0.14	0.05	−0.24	0.32

IV, Independent Variable (Variance); M, Mediating Variable (Normative Expectation); DV, Dependent Variable (Contribution). SE (for Standard Error) is the posterior standard deviation. pme is the proportion of effect that is mediated.

In the mediation analysis, the range of empirical expectations is from 0 to 20, while normative expectations range from −1 to 1. The coefficient of variance for empirical expectations (−0.30) is greater than that for normative expectations (−0.03) due to the varying value ranges (Figure 7). When the normative expectations variable is adjusted to a range of −10 to 10, the coefficient of variance for normative expectations becomes −0.32, which is similar to that for empirical expectations. Similarly, the coefficient for normative expectations on behavior (0.30) is greater than that for empirical expectations (0.12), due to their varying value ranges. When we adjust the range of the normative expectations to −10 to 10, the coefficient for behavior drops to 0.03, which is significantly smaller than that of empirical expectations on behavior (0.12).

In summary, the indirect effects of empirical expectations (a_1_ × b_1_ = −0.04, 95% CI: −0.10, 0.02) and normative expectations (a_2_ × b_2_ = −0.01, 95% CI: −0.06, 0.04) were not significant, suggesting that empirical expectations and normative expectations did not mediate between variance and contributions. Hypothesis 3 was not supported (i.e., Social norms play a mediating role in the relationship between variance and cooperative behavior).

## 5 Discussion

Our results indicate that given the same mean, the larger the variance in others’ contributions, the lower the contributions, empirical and normative expectations. Given the same mean for the three distributions, a larger variance means that there is a smaller minimum and a larger maximum contribution. This shows that, in addition to the mean, the minimum contribution is an important factor influencing cooperative behavior and social norms. Our study replicates Hartig et al.’s (2015) finding that people trended to follow the bad example of a low contributor when individual contributions were available in a one-shot linear public goods game. Our results are also consistent with Cheung’s (2014) finding that an individual had the highest contribution when others contributed equally and with experiments on group composition by Gächter and Thöni (2005) and De Oliveira et al. (2015) showing that homogeneous groups of non-selfish players had higher initial and overall contributions in repeated public goods games.

Our findings may enhance understanding of previous research on the use of punishment. Kirchkamp and Mill (2020) investigated how conditional cooperation changes when a participant can be punished or has the right to punish others. They found that the possibility of being punished increased the strength of conditional cooperation and the number of free riders, while the possibility of punishing others generally promoted cooperation. Consistent with several other studies (Fischbacher et al., 2001; Burlando and Guala, 2005; Kocher et al., 2008; Herrmann and Thöni, 2009; Hartig et al., 2015), conditional cooperators typically contribute less than perfect conditional cooperation. If these cooperators receive only average contributions, they will punish others by contributing less, which can reduce the payoffs of all group members and result in lower levels of cooperation. Therefore, disclosure of individual contributions is necessary when introducing peer punishment.

Our findings that both negative and positive deviations from the mean are considered less socially appropriate are consistent with Kölle and Quercia (2021). This suggests that people dislike others who behave too selfishly, and they also dislike others who behave too altruistically. In particular, the fact that behaving too altruistically is considered less socially appropriate may be due to the following reasons. First, people may believe that exploiting others is unacceptable, but being exploited by others should also be avoided. Second, people may believe that behaving too altruistically reflects poorly on others and arouses suspicion or resentment, which could lead to antisocial punishment (Herrmann et al., 2008).

Our finding that empirical expectations have a much stronger effect on cooperation than normative expectations may further explain why the level of cooperation is below the social optimum. This highlights the importance of empirical expectations in sustaining cooperation. Consistent with the Bicchieri’s argument (Bicchieri, 2006, 2016), individuals render their empirical expectations on the norm compliance of others a crucial element for their decision-making.

Previous studies have suggested that social preferences play an important role in norm compliance (Fehr and Schurtenberger, 2018). For example, people have an intrinsic desire for fairness, and deviating from social norms that are perceived as fair creates psychic costs of compliance (Fehr and Schmidt, 1999; Bolton and Ockenfels, 2000; López-Pérez, 2008). This means that social norms substantially influence people’s motivation by affecting what is perceived as equitable, while the intrinsic desire for equity ensures compliance with the norm. Again, another reason why people comply with the norm might be that people have reciprocal preferences. Based on reciprocal preferences, people trend to reward kind intentions with kindness and to punish unkind intentions. But this requires defining what constitutes kind and unkind behavior. Kind intentions are usually defined based on the normative notions of fairness, and what is perceived as fair may be perceived as kind. For an individual with reciprocal preferences, failure to reciprocate to kind behavior or to punish unkind behavior imposes psychic costs of noncompliance with norms (Rabin, 1993; Dufwenberg and Kirchsteiger, 2004; Falk and Fischbacher, 2006).

However, although intrinsic motives of individuals are assumed to remain stable across contexts, this does not imply that what is defined as fair/kind is stable across contexts. Our study reveals that the definition of fair or kind can vary across distributions. In the zero variance condition, all three group members contributed 10 MU, and a contribution of 10 MU was perceived as fair and kind (in fact, the empirical expectation was 7.2 MU); in the medium variance condition, the minimum was 5 MU, what was perceived as fair and kind was likely to be a contribution of less than 10 MU (in fact, the empirical expectation was 5.9 MU), because people tend to follow the bad example; similarly, in the large variance condition, the minimum was 0 MU, what was perceived as fair and kind could be a contribution further below 10 MU (in fact, the empirical expectation was 4.2 MU). In general, the heterogeneity of others’ contributions affects social norms by influencing what is fair and kind, and social norms serve as reference points to influence people’s cooperative behavior.

This study has limitations. Firstly, a relatively large sample is needed to analyze the mediating effect. Due to the sample limitation, this paper presents a preliminary exploration of mediating effect of social norms between variance and cooperative behavior. In future studies, the effect should be examined in a much larger sample (with *a priori* power analysis) before drawing conclusions. Additionally, this paper solely examines the impact of variance on social norms and cooperative behavior. However, investigating the interactive influence of variance and mean on social norms and behavior is an intriguing topic for further exploration.

## 6 Conclusion

In this paper, we investigate the relationship between the variance of other group members’ contributions, social norms and cooperative behavior using a classic linear public goods game. The following results are found. First, given the same mean, the greater the variance of a participant’s peers’ contributions, the lower their contributions, empirical expectations, and normative expectations, even though the behavior considered most socially appropriate was the same for different distributions. Second, both negative and positive deviations from the mean were considered less socially appropriate, suggesting that people dislike others who behave too selfishly, and they also dislike others who behave too altruistically. Third, empirical expectations had a much stronger effect on cooperative behavior than normative expectations. This highlights the importance of empirical expectations in sustaining cooperation. Fourth, although there was a strong relationship between variance, social norms, and cooperative behavior, the mediating effect of social norms was not significant. Finally, some of the findings regarding behavior type are also interesting. Despite the very different behavior of free riders and cooperators, their normative expectations were similar, that is, they all shared the same beliefs about how people ought to behave. The empirical expectations of free riders were much lower than those of cooperators, which did not differ from their actual contributions. This shows that empirical expectations change easily with the situation, while normative expectations are more stable.

Our research may have potential policy implications. Specifically, it suggests that conditional cooperators are more likely to follow negative examples. This could be valuable for managing teams, designing incentives, and providing feedback. It could also provide useful insights for interventions by changing the beliefs on others’ cooperation. However, we suggest thorough replication studies and field studies before drawing stronger conclusions.

## Data availability statement

The datasets presented in this study can be found in online repositories. The names of the repository/repositories and accession number(s) can be found in the article/supplementary material.

## Ethics statement

The studies involving humans were approved by the Ethics Committee of College of Economics, Shandong University. The studies were conducted in accordance with the local legislation and institutional requirements. The participants provided their written informed consent to participate in this study.

## Author contributions

GW: Conceptualization, Formal analysis, Methodology, Writing – original draft, Funding acquisition. JL: Conceptualization, Funding acquisition, Methodology, Project administration, Writing – original draft. WW: Data curation, Formal analysis, Methodology, Writing – original draft. YW: Data curation, Formal analysis, Writing – original draft. JW: Formal analysis, Software, Writing – review & editing.

## Footnotes

^1^In this study, empirical expectations and normative expectations are used to characterize social norms.

^2^Between-subjects and within-subjects designs are important experimental designs. In between-subjects designs, subjects can be randomly assigned to one of two or more groups. Comparing the groups informs us about the treatment effects on the subjects. These effects at the treatment level indicate differences between the participants. In a within-subjects design, each participant experiences every condition of the independent variable. This design controls for individual differences and often requires fewer participants. The within-subjects design can also be called a repeated-measures design.

^3^Repeated ANOVA is the common method used to analyze the data from the within-subjects design. In the study, the contributions (3 levels: zero, medium, large variance), empirical expectations (3 levels: zero, medium, large variance) and normative expectations (3 levels: zero, medium, large variance) are repeated.

^4^The T represents the T-statistic of the paired samples t-test. We did not correct them using Bonferroni’s method.
